# Effects of angiotensin converting enzyme gene polymorphism on hypertension in Africa: A meta-analysis and systematic review

**DOI:** 10.1371/journal.pone.0211054

**Published:** 2019-02-14

**Authors:** Hayelom Gebrekirstos Mengesha, Pammla Petrucka, Cara Spence, Tadesse Bekele Tafesse

**Affiliations:** 1 Department of Pharmacy, College of Medicine and Health Sciences, Adigrat University, Adigrat, Ethiopia; 2 University of Saskatchewan, College of Nursing, Saskatoon, SK, Canada, Adjunct Nelson Mandela African Institute of Science and Technology, Arusha, Tanzania; 3 Univeristy of Saskatchewan, International Research Specialist, International Office, Saskatoon, SK, Canada; 4 School of Pharmacy, College of Health and Medical Sciences, Haramaya Univeristy, Harar, Ethiopia; GeneDx, UNITED STATES

## Abstract

**Background:**

Hypertension is dramatically increasing in Africa with evidence of increased severity and resistance to treatment. Although angiotensin converting enzyme gene polymorphism is associated with higher prevalence of hypertension, the evidence is inconclusive on its influence on the emerging pattern in Africa. This meta-analysis is conducted to pool the available evidence to inform future research and interventions.

**Methods:**

Articles published through May 2018 were systematically searched in PubMed, Scopus and EMBASE databases. Studies were assessed for inclusion by two independent researchers. Six models were used to assess the effect of angiotensin converting enzyme deletion-insertion gene polymorphism. Heterogeneity and publication bias were tested and sensitivity analysis was carried out. Odds ratio and 95% confidence intervals were measured for pooled effect. Both random effect and fixed effect models were used, whilst the frequency of DD, II and DI genotypes were computed and compared.

**Result:**

Patients with D allele were 1.49 times more likely to develop essential hypertension compared with patients who carry the I allele (OR:1.49; CI:1.07, 2.07). Similarly, patients who had homozygous co-dominance genotype DD (i.e., DD vs II) were at a 2.17 times higher risk of essential hypertension compared to the co-dominant genotype II (OR:2.17, CI:1.79, 3.18), dominant model (I.e., DD+ID vs II) (OR:1.48; CI:1.03, 2.12), and recessive model (OR:1.64; CI:1.03, 2.61). On subgroup analysis, participants from Sub-Saharan Africa were more genetically susceptible to hypertension compared to their North Africa counterparts. There was no publication bias found, but there was high to moderate heterogeneity.

**Conclusion:**

ACE I/D polymorphism is associated with essential hypertension in Africa in the allele contrast model, as well as the dominant, recessive and homozygous codominance model. On subgroup analysis, ACE I/D was associated with essential hypertension in patients from Sub-Saharan Africa but not in North Africa. A future large scale study, which includes different ethnic groups, is recommended.

## 1. Introduction

Globally, hypertension prevalence among adults aged 18 years and older was reported as 22.2%; however, prevalence in Africa was highest at 29.6% [1]. According to Kearney *et al*, 75% of deaths in Sub-Saharan Africa (SSA) by 2020 will be attributable to hypertension [2]. Studies indicated that, in African patient populations, hypertension is more severe, resistant to treatment, and likely to lead to immediate end organ damage and premature death [3–6]. Further, it was found that, despite lifestyle adjustment and treatment options, the hypertension burden is increasing and globally is expected to reach 1.56 billion adults by 2025 with disproportionate representation in SSA [2].

Growing hypertension prevalence and related complications are attributed to environmental and genetic factors [7– 9]. The heritability of hypertension ranges from 24% to 50% [8,9]. While 12 causative genes have been identified for hypertension, essential hypertension has been associated with a complex relationship of multiple polymorphisms in different genes. More than 60 single nucleotide polymorphisms (SNPs) have been reported in European populations [10,11], African Americans [12], and Asians [13,14]. According to these studies, known loci account for only 2.5% of the phenotypic variance for systolic and diastolic blood pressure BP [12]. Research is being done to trace missing heritability of hypertension by investigating rare and structural sequence variants, epigenetics, and investigating gene interactions [12]. Genomic wide association and sequencing analysis are devised to help in analysis of missing heritability.

There are different genetic determinants involved in the pathogenesis of hypertension. The renin angiotensin aldosterone system (RAAS) is one of the most important pathways in the pathogenesis and management of hypertension. Angiotensin converting enzyme (ACE) is among the proteins involved in this pathway effecting salt retention, water balance and blood vessels; hence, it controls blood pressure. Drugs which inhibit this enzyme are effective in treatment of hypertension; however, these drugs are not first-line treatment in patients of African and African American origin [15]. Genetic polymorphism is associated with the diminished effect of angiotensin converting enzyme inhibitors [16].

Angiotensin converting enzyme is encoded by the ACE gene, which is found in 17q23 and encoded by a 21 kb gene that consists of 28 exons and 25 introns. The insertion/deletion (I/D) polymorphism of ACE is characterized by the presence (insertion) or absence (deletion) of a 28 bp Alu repeat sequence in intron 16 producing three genotypes (II homozygote, ID heterozygote and DD homozygote). Although the I/D polymorphism is located on a non-coding region (i.e., intron) of the ACE gene, several investigators found that the *D* allele is related to increased activity of ACE in serum [17]. In this gene, ACE I/D polymorphism is associated with essential hypertension in studies conducted in Africa [18–21]. However, there is no pooled evidence and there are conflicting findings on the effect of ACE I/D in essential hypertension studies conducted in Africa. This divergence is seen in a few studies in Africa which found association between this polymorphism and essential hypertension [19–21]; whereas others did not yield similar findings [22–25]. Therefore, the aim of this meta-analysis is to pool the available evidence and provide insights into the potential linkage(s) between the ACE gene and its effect on treatment and pathogenesis of hypertension in individuals of African origin.

## 2. Methods and materials

The study is conducted according to the HuGENet HuGE Review Handbook, Version 1.0 [26] and the PRISMA checklist (S1 Table).

### 2.1 Search strategy

We searched PubMed, Embase and Scopus databases for articles published through May 2018 which investigated the association between ACE I/D gene polymorphism and the risk of essential hypertension. The search was limited to English language articles. The search terms used were as follows: (Hypertension OR “essential hypertension” OR “high blood pressure OR raised blood pressure”) AND (“angiotensin-converting enzyme” OR ACEI) AND (insertion/deletion OR ACE I/D) AND “gene polymorphism” AND Africa (S1 Database). We also reviewed the reference lists of included studies and reviews. If there was repetition in the retrieved studies, we chose the one with most complete analysis.

### 2.2 Inclusion and exclusion criteria

We included studies which were case-control and used hypertension as an outcome of interest. Studies were excluded if they were case reports/editorials/reviews or multiple publications of the same dataset. Studies were excluded if they did not report frequency of all alleles and genotype, and relationship between other genes and essential hypertension and studies which investigated the role of ACE I/D in other diseases.

### 2.3 Data extraction and study selection process

Variables were extracted using checklist from all studies. The following study characteristics were recorded in our study: first author’s surname, year of publication, ethnicity of study population, number of cases and controls for ACE I/D genotype. Gene, polymorphism, and frequencies of D and I allele were computed for case and control groups from the corresponding genotype distribution. Two authors independently conducted the data extraction and synthesis, with any discord resolved through discussion.

### 2.4 Statistical analysis

Odds ratio (OR) and 95% confidence interval (CI) was used to measure the strength of the association between ACE I/D gene polymorphism and essential hypertension across studies. Heterogeneity was measured by I^2^ statistics. *Q* statistic (significance level at *p*<0.05) was also determined. I^2^ <25% minimal heterogeneity, 25–75% moderate heterogeneity and >75% extreme heterogeneity. The pooled ORs were computed using either fixed-effects model or random-effects model. Random effect model was used if the studies had heterogeneity and if there no fixed effect model was used.

Different genetic models were used to compare the genotypes and alleles. Allele contrast model (D vs I), recessive model, dominant model, overdominance, as well as homozygous and heterozygous co-dominance models were used. Forest plot was conducted for all models to identify the individual and pooled effect of all studies included in this meta-analysis. Agreement of the genotype frequencies to Hardy–Weinberg equilibrium (HWE) was tested using chi square (HWE significance level at *p*<0.05). A sensitivity analysis was conducted by subsequent omission of individual studies and looked for the difference in change of OR and its associated significance. Potential publication bias was assessed by Egger’s test (significance level at *p*<0.05). All analyses were conducted using the Web tool META-Genyo, Version 12.0. [27].

## 3. Results

### 3.1 Search history

**Fig 1:**
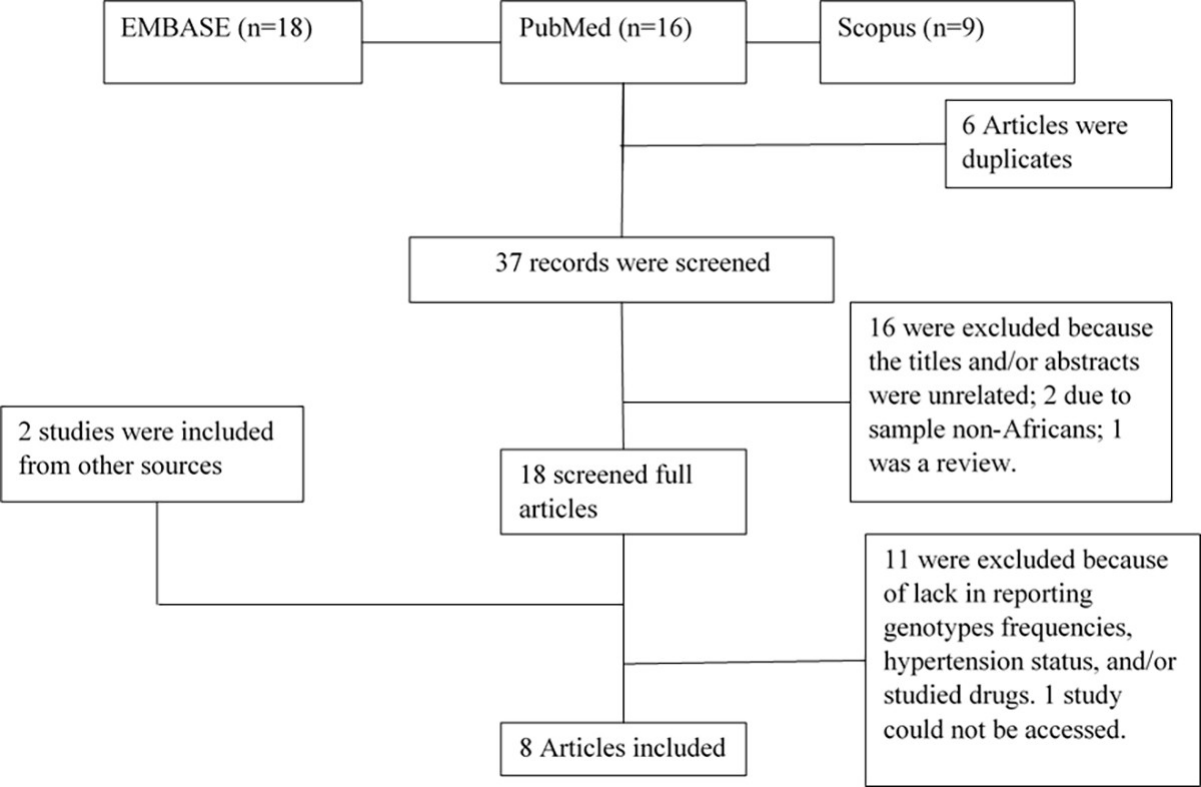
Flow diagram for the study selection process for inclusion in this meta-analysis.

The database search of three databases produced 43 articles to be assessed for inclusion or exclusion from the meta-analysis. Of these, 6 articles were duplicates, whilst 16 were excluded because title and/or abstract did not meet the inclusion criteria. After further screening, 8 studies were found to fulfil the inclusion criteria and were considered in this meta-analysis (Fig 1).

### 3.2 Study characteristics

Eight studies, including 4 from sub-Saharan Africa (Gabon, Nigeria, Ghana, South Africa) and 4 from North Africa (three Egypt and one Tunisia), met the inclusion criteria. Sample size ranged from 61 to 1224. Mean age (±sd) of the studied populations were between 45 (±8.2) and 62.8 (±9.9) for cases and 31.9 (±10.27) to 54.4 (±9.5) for controls. Mean (±sd) blood pressure for the participants were different among the included studies. One study did not report blood pressure [23]. The mean SBP/DBP was highest in a study conducted in Egypt [21] reporting a BP level of 164.9 (±23.2)/99.1 (±11.6). Sample size was largest (1224) in a study conducted by Mary et al. [19] and lowest (61) in a study by Imen et al [23] (Table 1).

**Table 1:** Study characteristics of the studies included to the meta-analysis.

		Age mean (+SD)		Hypertension(SBP/DBP)mean(+SD)		
Reference	No case/control	Cases	Control	Case	Control	Cases/Controls
Mary et al 2014	612/612	51.3(13.76)	31.9(10.27)	161.1(13.26)/93.2(13.7)	116.7(9.19)/72.18(8.4)	27.48(5.81)/23.32(5.83)
Zarouk WA et al 2012	40/21	57.1(8.9)	46.4(14.3)	164.9(23.2)/99.1(11.6)	126.1(8.7)/78(7.1)	NA
Imen A et al 2010	39/22	62.8(9.9)	54.4(9.5)	NA	NA	28.8(+5.9)/25.8(3.6)
Ndong AGR et al 2018	95/37	58.37(11.4)	40.9(13.1)	155.8(29.6)/85(2.61)	117.36(14.1)/72(14.5)	NA
Naglaa RAR et al 2012	110/93	45(8.2)	42(7.3)	147.8(13.5)/95.6(6.3)	131.4(13.4)/82(7.2)	28.8(4.5)/28.02(11)
Daméhan T et al 2015	202/204	51(10)	49.5(13.54)	160(20.66)/95(11.87)	120(11.47)/70(8.24)	27(6.48)/23(4.9)
Hayet et al 2013	388/425	55.19(9.9)	52.1(13.1)	151.54(19.3)/87.6(11.1)	118.68(10.59)/71.53(6.7)	31.14(9.9)/27.26(4.91)

### 3.3 Genotype frequency

All studies had higher frequencies of the DD genotype compare to DI and II genotypes in the hypertensive group; whereas, in the control group, the distribution varied depending on the study. There was no violation of HWE in any included studies (Table 2).

**Table 2:** Genotype studies of the included studies.

Author	Ethnicity	DD.cases	DI.cases	II.cases	DD.controls	DI.control	II.controls	HW^#^-P.value	HW-adjusted.P.value
Mary et al	SSA*	192	171	44	153	190	57	0.871	0.957
Zarouk WA et al	NA^$^	24	14	2	7	8	6	0.278	0.643
Imen A et al	NA	19	16	4	8	12	2	0.402	0.643
Ndong AGR et al	SSA	55	33	7	15	19	3	0.368	0.643
Naglaa RAR et al	NA	34	59	17	25	52	16	0.214	0.643
Hayet et al	NA	173	176	39	205	180	40	0.957	0.957
Scott et al	SSA	38	74	14	16	24	11	0.722	0.957

* Sub-Saharan Africa

$ North Africa

# hardy Weinberg

### 3.4 Meta-analysis

There was no publication bias in all studies and models considered in this study. There was heterogeneity among the studies in the included models where the p-value for Q was <0.05 and I^2^ >50%. The sources for the heterogeneity might be in the difference in sample size and difference in the geographical areas where the studies were conducted (i.e., SSA vs NA) which relate to ethnicity. ACE deletion/insertion polymorphism was significantly associated with essential hypertension in the allele contrast model (P = 0.01) showing significant heterogeneity (P = 0.0001) and no publication bias (P = 0.32); the recessive model (P = 0.03) showing significant heterogeneity (P = 0.0001) and no publication bias (P = 0.46); and the dominant model (P = 0.032 and with non-significant heterogeneity (P = 0.12) or publication bias (P = 0.28); and the homozygote codominant model (P = 0.018) with significant heterogeneity (P = 0.008) and no publication bias (P = 0.30). In the remaining models, the ACE I/D polymorphism gene did not have significant association with essential hypertension (Table 3).

**Table 3:** Genetic association values with test result for publication bias.

Model	OR	95% CI	P-value			Egger's test
Heterogeneity tests	tau^2^	H	I^2^%	Q	P-value	
Allele contrast	1.49	1.07,2.07	0.01			0.32
Heterogeneity tests	0.17	2.5	83	43.51	0.0001	
Recessive model	1.63	1.03,2.6	0.03			0.46
Heterogeneity tests	0.33	2.54	84	45.34	0.0001	
Dominant model	1.47	1.03,2.11	0.032			0.28
Heterogeneity tests	0.091	1.22	38	11.4	0.12	
Over dominant model	0.75	0.5,1.11	0.15			0.66
Heterogeneity tests	0.22	2.19	79	33.62	0.0001	
Homozygote codominant model	1.83	1.1,3.03	0.018			0.30
Heterogeneity tests	0.28	1.6	62	18.84	0.008	
Heterozygote codominant model	1.54	0.97,2.44	0.06			0.5
Heterogeneity tests	0.31	2.38	0.82	39.82	0.0001	

### 3.5 Quantitative synthesis by forest plot with OR and 95% confidence interval

**Fig 2:**
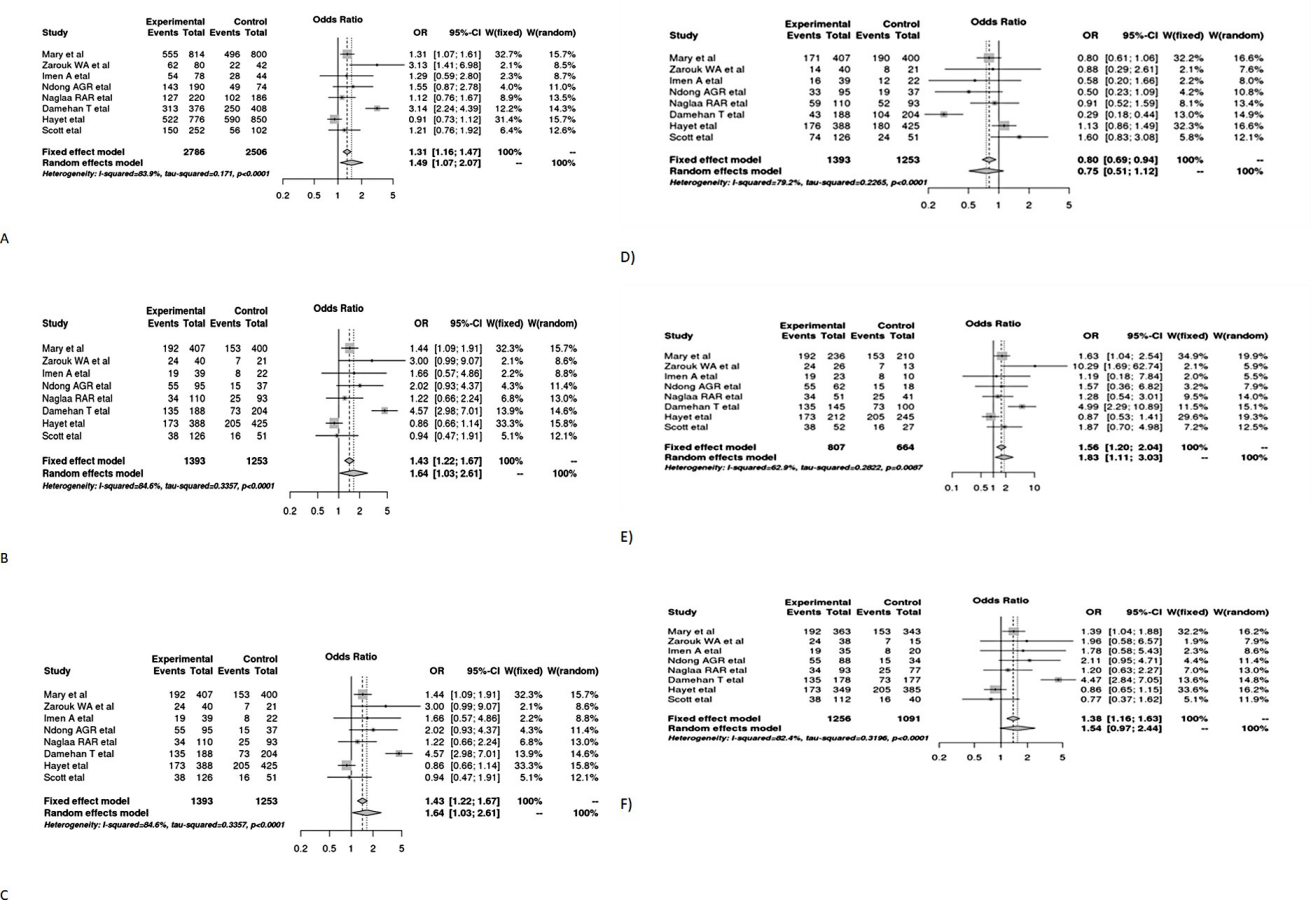
Forest plot for the effect of ACE I/D gene polymorphism in different models on the association with essential hypertension on across studies conducted in Africa. A) Allele contrast model B) recessive model C) dominant model D) over dominant model E) homozygous co-dominant model F) heterozygous co-dominant model.

The forest plot for the six models shows the relationship of each study with essential hypertension in Africa, as well as an overall pooled estimate. The figures show that essential hypertension was associated with polymorphism in the allele contrast, recessive model (DD vs ID+II), dominant model (DD+ID vs II), and homozygote co-dominant model (DD vs II). Since there was heterogeneity across the studies, the random effect model was used to estimate the odds ratio. Patients with D allele were 1.49 times higher to develop essential hypertension compared with those who carry the I allele (OR:1.49; 95% CI:1.07,2.07). Similarly, patients with homozygous codominance genotype (DD) are at a 2.17 times higher risk of essential hypertension compared to those with the co-dominant genotype II (OR:2.17; 95% CI:1.79,3.18), dominant model (DD+ID vs II) (OR:1.48; 95% CI:1.03,2.12) and recessive model (OR:1.64; 95% CI:1.03,2.61) (Fig 2).

### 3.6 Sensitivity and subgroup analysis

**Fig 3:**
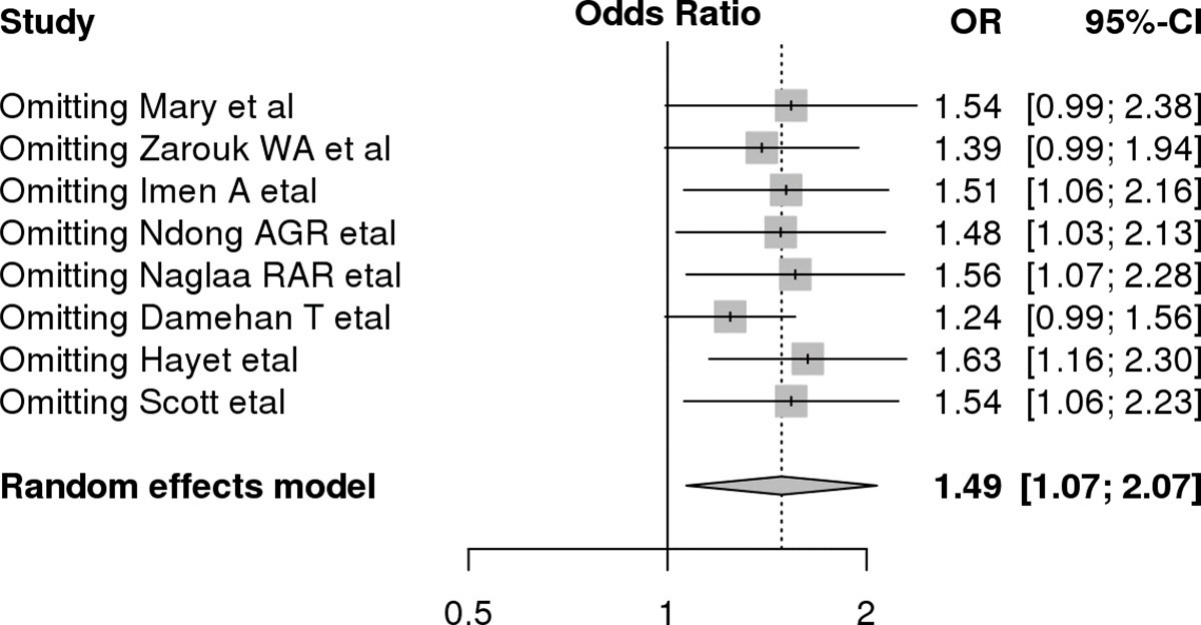
Sensitivity analysis for allele contrast model which shows the variation or change in effect estimate based on omission of studies.

A subsequent sensitivity analysis by omission of studies was done to check for instability and change in significance of the effect estimate. After each study was excluded from the current meta-analysis, as can be seen from the graph, there was no significant shift or change in the level of significance and odds ratio. Studies by Mary et al. [19], Damehan, et al. [18], and Zarouck, et al. [21] caused a slight shift in the OR and significance, but this shift was not considered a large due to the lower CI being 0.99, the small number of studies in this meta-analysis, and the HWE not being violated (Fig 3).

### 3.7 Subgroup analysis by self-reported ethnicity

On subgroup analysis by classifying studies into North Africa [21–24] and SSA [18, 19, 25, 28], the findings showed that ACE I/D polymorphism was associated with essential hypertension in patients from SSA. Significant association was shown in the dominant model for SSA (OR:1.65; 95% CI: 1.18,2.29), allele contrast model (OR:1.68; 95% CI:1.04,2.7), and homozygote co-dominant model (DD vs. II) (OR:2.07; 95% CI: 1.45,2.94). Heterogeneity has also been reduced compared to the overall analysis **(**Table 4).

**Table 4:** Subgroup analysis of patients by reported ethnicity (random model effect is used).

Model	Group	OR(95% CI)	P-value	I^2^
Allele contrast	Overall	1.47(1.06,2.02)	0.01	0.83
	NA	1.25(0.82,1.89)	0.28	0.67
	SSA	1.68(1.04,2.7)	0.03	0.85
Recessive model	Overall	1.63(1.03,2.6)	0.036	0.84
	NA	0.99(0.78,1.26)	0.97	0.51
	SSA	1.89(0.95,3.84)	0.06	0.87
Dominant model	Overall	1.37(1.07,1.75)	0.011	0.38
	NA	1.07(0.74,1.57)	0.68	0.45
	SSA	1.65(1.18,2.29)	0.027	0.046
Overdominant model	Overall	0.74(0.5,1.11)	0.15	0.79
	NA	1.03(0.82,1.31)	0.75	0
	SSA	0.64(0.32,1.28)	0.21	0.87
Homozygote codominant	Overall	1.83(1.1,3.03)	0.01	0.63
	NA	1.44(0.64,3.22)	0.36	0.56
	SSA	2.07(1.45,2.94)	0.0046	0.52

## 4. Discussion

In this study we hypothesized that the effect of ACE in the management and treatment of hypertension is highly correlated with genetics.

Angiotensin converting enzyme inhibitors are not effective first-line treatments in people of African origin compared to Caucasians [15]. In addition, hypertension in Africa is more severe and resistant to treatment [3–6]. While there are different environmental factors that play a role for the raised BP, studies have shown genetics made a significant contribution [8–9]. RAAS related genetic polymorphism is among the most widely studied pathway for the management and control of hypertension. ACE I/D polymorphism is one of the most thoroughly studied polymorphisms in Africa. Due to inconsistencies among different studies, we pooled the available evidence in this meta-analysis.

This study found that ACE I/D polymorphism is associated with hypertension in two models, specifically the allele contrast model, and the recessive, dominant and homozygous co- dominant model. This study showed that the dominant D allele is important for the increased expression of ACE activity [29]. This finding is consistent with a meta-analysis done in China [30] but contradicts a recent meta-analysis [31]. There were differences in some studies including diseases apart from hypertension, as well as differences in terms of the various studies’ sample sizes and methodologies. There was significant heterogeneity which may relate to differences in the backgrounds/ethnicities of our study population (i.e., North Africa and SSA), small sample sizes, or the limited number of studies. In conclusion, the findings suggest a large scale multi-ethnic study is needed to further elucidate the effect(s) of ACE I/D polymorphism on hypertension.

Over-dominant model [DI vs DD+II] was not significant in determining essential hypertension. This could be because of the traits that patients acquired from both homozygous and heterozygous alleles might be abolished each other. Consequently, it could have a null effect in the pathogenesis of hypertension.

On subgroup analysis, patients from Sub-Saharan Africa showed higher susceptibility to hypertension than patients from North Africa. Previously ACE inhibitors were found ineffective as first-line medications for the management of hypertension in Africa [15], although the reason remained elusive. Although different factors, such as low renin, low nitric oxide, and high salt retention, were found to be related, these determinants explained a small fraction of the variance [15]. Genetic polymorphism remains under investigated in the African population. In this study, we found significant association on subgroup analysis in dominant, allele contrast model and homozygous model which shows the D allele is related with increased activity of ACE. However, this finding should be interpreted cautiously as the subgroup analysis included a small number of studies and limited sample size. Therefore, these evidence deficits warrant further investigations in Africa, with consideration for East Africa where no studies were included in the meta-analysis.

### 4.1 Limitations and strengths of this study

To the best of our knowledge, this is the first meta-analysis which specifically assesses the effect of ACE I/D polymorphism on essential hypertension in Africa, as well as conducting subgroup analysis which opens the door for further research. Although, there was no violation of HWE and publication bias, there was significant heterogeneity which could relate to variation in study sample size, populations, and ethnicity of participants.

### 4.2 Conclusion

ACE I/D polymorphism is associated with essential hypertension in the allele contrast model, dominant allele contrast model, and homozygous codominance model in Africa. On subgroup analysis, ACE I/D was found to be associated with essential hypertension in patients from Sub-Saharan Africa but not North Africa.

## Supporting information

S1 Table PRISMA 2009 checklist. (DOC)

S1 Database Sample database search for PubMed. (DOCX)

S1 Articles Articles used to draw the conclusion. (DOCX)

## References

[1] World Health Organization. World Health Statistics Reports on Global Health Goals for 194 Countries. 2015. Available at: http://cdrwww.who.int/mediacentre/news/releases/2015/world-healthstatistics-2015/en

[2] Kearney PM, Whelton M, Reynolds K, Muntner P, Whelton PK, et al Global burden of hypertension: analysis of worldwide data. Lancet. 2005; 365: 217–223. 10.1016/S0140-6736(05)17741-1 15652604

[3] Adigun AQ, Ishola DA, Akintomide AO, Ajayi AAL. Shifting trends in the pharmacologic treatment of hypertension in a Nigeria tertiary hospital: a real-world evaluation of the efficacy, safety, rationality and pharmaco-economics of old and newer antihypertensive drugs. J Hum Hypertens. 2003; 17:277–285. 10.1038/sj.jhh.1001538 12714973

[4] Brewster LM, van Montfrans GA, Kleijnen J. Systematic review: antihypertensive drug therapy in black patients. Ann Intern Med. 2004; 141:614–627. 15492341 10.7326/0003-4819-141-8-200410190-00009

[5] Lewington S, Clarke R, Qizilbash N, Peto R, Collins R. Age-specific relevance of usual blood pressure to vascular mortality: a meta-analysis of individual data for one million adults in 61 prospective studies. Lancet. 2005; 360:1903–13. 10.1016/s0140-6736(02)11911-8 12493255

[6] Opie LH, Seedat YK. Hypertension in sub-Saharan African populations. Circulation. 2005; 112:3562–3568. 10.1161/CIRCULATIONAHA.105.539569 16330697

[7] Rodrigues SL, Baldo MP, Machado RC, Forechi L, Molina Mdel C, Mill JG. High potassium intake blunts the effect of elevated sodium intake on blood pressure levels. J Am Soc Hypertens. 2014; 8:232–238. 10.1016/j.jash.2014.01.001 24524886

[8] Kupper N, Ge D, Treiber FA, Snieder H. Emergence of novel genetic effects on blood pressure and hemodynamics in adolescence: The Georgia Cardiovascular Twin Study. Hypertens. 2006; 47:948–954. 10.1161/01.HYP.0000217521.79447.9a 16567584

[9] van Rijn MJ, Schut AF, Aulchenko YS, Deinum J, Sayed-Tabatabaei FA, Yazdanpanah M, et al Heritability of blood pressure traits and the genetic contribution to blood pressure variance explained by four blood-pressure-related genes. J Hypertens. 2007; 25(3):565–570. 10.1097/HJH.0b013e32801449fb 17278972

[10] Wellcome Trust Case Control Consortium. Genome-wide Association study of 14,000 cases of seven common diseases and 3000 shared controls. Nature. 2007; 447:661–678. 10.1038/nature05911 17554300 PMC2719288

[11] Ehret GB, Munroe PB, Rice KM, Bochud M, Johnson AD, Chasman DI, et al Genetic variants in novel pathways influence blood pressure and cardiovascular disease risk. Nature. 2011; 478:103–109. 10.1038/nature10405 21909115 PMC3340926

[12] Franceschini N, Fox E, Zhang Z, Edwards TL, Nalls MA, Sung YJ, et al Genome-wide association analysis of blood-pressure traits in African-ancestry individuals reveals common associated genes in African and non-African populations. Am J Hum Genet. 2013; 93:545–554. 10.1016/j.ajhg.2013.07.010 23972371 PMC3769920

[13] Kato N, Takeuchi F, Tabara Y, Kelly TN, Go MJ, Sim X, et al Meta-analysis of genome-wide association studies identifies common variants associated with blood pressure variation in east Asians. Nat Genet. 2011; 43:531–538. 10.1038/ng.834 21572416 PMC3158568

[14] Kelly TN, Takeuchi F, Tabara Y, Edwards TL, Kim YJ, Chen P, et al Genome-wide association study meta-analysis reveals transethnic replication of mean arterial and pulse pressure loci. Hypertens. 2013; 62:853–859. 10.1161/HYPERTENSIONAHA.113.01148 PMC3972802 24001895

[15] Brewster LM, Seedat YK. Why do hypertensive patients of African ancestry respond better to calcium blockers and diuretics than to ACE inhibitors and β-adrenergic blockers? A systematic review. BMC Medicine. 2013; 11:141 10.1186/1741-7015-11-141 23721258 PMC3681568

[16] Arnett DK, Davis BR, Ford CE, Boerwinkle E, Leiendecker-Foster C, Miller MB, et al Pharmacogenetic association of the angiotensin-converting enzyme insertion/deletion polymorphism on blood pressure and cardiovascular risk in relation to antihypertensive treatment: the genetics of hypertension-associated treatment (GenHAT) study. Circ. 2005; 111(25):3374–83. 10.1161/CIRCULATIONAHA.104.504639 15967849

[17] Staessen JA, Wang JG, Ginocchio G, Petrov V, Saavedra AP, Soubrier F. The deletion/insertion polymorphism of the angiotensin converting enzyme gene and cardiovascular renal risk. J Hypertens. 1997; 15: 1579–1592. 9488209 10.1097/00004872-199715120-00059

[18] Daméhan T, Kologo JK, Karou SD, Yaméogo VN, Bisseye C, Djigma FW, et al Renin-Angiotensin system genes polymorphisms and essential hypertension in Burkina Faso, West Africa. International Hypertens. 2015; 979631. 10.1155/2015/979631 PMC4553326 26351579

[19] Mary EK, Anumudu CI, Kumar PL. Insertion/deletion polymorphism of the angiotensin-converting enzyme gene and the risk of hypertension among residents of two cities, South-South Nigeria. Adv Biomed Res. 2014; 3: 118 10.4103/2277-9175.133184 24949289 PMC4063107

[20] Nermin Z, Shaker D, Abdelaal A, Aref W. Angiotensin-converting enzyme gene polymorphisms and hypertension in occupational noise exposure in Egypt. Occupational and Environmental Health. 2014; 20(3):194–206. 10.1179/2049396714Y.0000000067 PMC4090884 25000107

[21] Zarouk WA, Hussein IR, Esmaeil NN, Raslan HM, Reheim HAA, Moguib O, et al Association of angiotensin converting enzyme gene (I/D) polymorphism with hypertension and type 2 diabetes. Bratisl Lek Listy 2012; 113(1):14–18. 22380495 10.4149/bll_2012_003

[22] Hayet S, Kabadou IA, Jemaa R, Feki M, Kallel A, Souheil O. G protein beta3 subunit gene C825T and angiotensin converting enzyme gene insertion/deletion polymorphisms in hypertensive Tunisian population. Clin Lab. 2013; 59(1–2):85–92. 23505911 10.7754/clin.lab.2013.111105

[23] Imen A, Nouira S, Abid A, Bouafif-Ben NA, Zorgati MM, Malouche D. Lack of association between renin-angiotensin system (RAS) polymorphisms and hypertension in Tunisian type 2 diabetics. La tunisie Medicale. 2010; 88(1): 38–41. 20415212

[24] Naglaa R, Abd-Raboh Neveen, Salah EDH, Manal LL, Samah EB. Association of angiotensin T235 polymorphism with risk of essential hypertension in Egyptian patients. International Journal of Cancer Research. 2012; 8(3):69–82.

[25] Ndong AGR, Obame-Engongra LC, Ovono AF. Correlation between the insertion-deletion polymorphism of the angiotensin conversion enzyme gene and hypertension among the Gabonse population. 2018; 12(2): 44–52.

[26] Little J, Higgins JPT. The HuGENet HuGE Review Handbook, version 1.0. http://www.hugenet.ca

[27] Martorell-Marugan J, Toro-Dominguez D, Alarcon-Riquelme ME, Carmona-Saez P, MetaGenyo: A web tool for meta-analysis of genetic association studies. BMC Bioinformatics. 2017; 18:563 10.1186/s12859-017-1990-4 29246109 PMC5732412

[28] Williams SM, Ritchie MD, Phillips JA, Dawson E, Prince M, Dzhura E, et al Multilocus analysis of hypertension: a hierarchical approach. Hum Hered. 2004; 57:28–38. 10.1159/000077387 15133310

[29] Li Y. (2011). Angiotensin–converting enzyme gene insertion/deletion polymorphism and essential hypertension in the Chinese population: a meta–analysis including 21,058 participants Intern Med J. 2012; 42(4):439–44. 21883781 10.1111/j.1445-5994.2011.02584.x

[30] Jing-Ren J, Horng-Jyh H, Chii-Yuan J, Kuo-Cheu Y, Shyh-Ming S.Angiotensin I converting enzyme gene polymorphism in Chinese patients with hypertension. Amer J Hypertensi. 1997;10(5):558–561. 10.1016/s0895-7061(97)00036-8 9160768

[31] Yandiswa YY, Eric VB, Tandi EM, Anastase D, Deirdre K, Eugene SCA, et al Genetic factors contributing to hypertension in African-based populations: a systematic review and meta-analysis. J Clin Hypertens. 2018; 20:485–495. 10.1111/jch.13225 PMC8031059 29520984

