# Supplementary material for: Effects of angiotensin converting enzyme gene polymorphism on hypertension in Africa: A meta-analysis and systematic review
Source: PLoS One. 2019 Feb 14;14(2):e0211054. doi: 10.1371/journal.pone.0211054 (PMC6375551; doi:10.1371/journal.pone.0211054)
Supplement: S1 Database — (DOCX) [file pone.0211054.s002.docx]

**Best matches for (Hypertension OR “essential hypertension” OR “high blood pressure OR raised blood pressure”) AND (“angiotensin-converting enzyme” OR ACEI) AND (insertion/deletion OR ACE I/D) AND “gene OR genepolymorphism” AND Africa*.:**

[Associations of **Angiotensin-Converting Enzyme** **Insertion/Deletion**, Angiotensin II Receptor A1166C, and Endothelial Nitric Oxide Synthase 4b/a **Gene** Polymorphisms With Pregnancy Hypertensive Disorders: A Meta-Analysis.](https://www.ncbi.nlm.nih.gov/pubmed/26119526)Gong FF et al. J Clin Hypertens (Greenwich). (2015)

[Genotypes and allele frequencies of **angiotensin-converting enzyme** (**ACE**) **insertion/deletion** **polymorphism** among Bahraini population with type 2 diabetes mellitus and related diseases.](https://www.ncbi.nlm.nih.gov/pubmed/22081332)Al-Harbi EM et al. Mol Cell Biochem. (2012)

[Case-control association study of polymorphisms in the angiotensinogen and **angiotensin-converting enzyme** **genes** and coronary artery disease and systemic artery **hypertension** in African-Brazilians and Caucasian-Brazilians.](https://www.ncbi.nlm.nih.gov/pubmed/27019433)Bonfim-Silva R et al. J Genet. (2016)

Switch to our new best match sort order

**Search results**

**Items: 18**

- Quoted phrase not found.

Select item 295488581.

[**ACE** **insertion/deletion** **polymorphism** is positively associated with resistant **hypertension** in Morocco.](https://www.ncbi.nlm.nih.gov/pubmed/29548858)

Abouelfath R, Habbal R, Laaraj A, Khay K, Harraka M, Nadifi S.

**Gene**. 2018 Jun 5;658:178-183. doi: 10.1016/j.**gene**.2018.03.028. Epub 2018 Mar 13.

PMID:

29548858

[Similar articles](https://www.ncbi.nlm.nih.gov/pubmed?linkname=pubmed_pubmed&from_uid=29548858)

Select item 278951972.

[The Effect of **ACE** **I/D** Polymorphisms Alone and With Concomitant Risk Factors on Coronary Artery Disease.](https://www.ncbi.nlm.nih.gov/pubmed/27895197)

Amara A, Mrad M, Sayeh A, Lahideb D, Layouni S, Haggui A, Fekih-Mrissa N, Haouala H, Nsiri B.

Clin Appl Thromb Hemost. 2018 Jan;24(1):157-163. doi: 10.1177/1076029616679505. Epub 2016 Nov 28.

PMID:

27895197

[Similar articles](https://www.ncbi.nlm.nih.gov/pubmed?linkname=pubmed_pubmed&from_uid=27895197)

Select item 261195263.

[Associations of **Angiotensin-Converting Enzyme** **Insertion/Deletion**, Angiotensin II Receptor A1166C, and Endothelial Nitric Oxide Synthase 4b/a **Gene** Polymorphisms With Pregnancy Hypertensive Disorders: A Meta-Analysis.](https://www.ncbi.nlm.nih.gov/pubmed/26119526)

Gong FF, Hu CY, Lu SS, Qian ZZ, Feng F, Wu YL, Yang HY, Sun YH.

J Clin Hypertens (Greenwich). 2015 Dec;17(12):954-62. doi: 10.1111/jch.12606. Epub 2015 Jun 29.

PMID:

26119526

[**Free Article**](https://www.ncbi.nlm.nih.gov/pubmed/26119526)

[Similar articles](https://www.ncbi.nlm.nih.gov/pubmed?linkname=pubmed_pubmed&from_uid=26119526)

Select item 250001074.

[**Angiotensin-converting enzyme** **gene** polymorphisms and **hypertension** in occupational noise exposure in Egypt.](https://www.ncbi.nlm.nih.gov/pubmed/25000107)

Zawilla N, Shaker D, Abdelaal A, Aref W.

Int J Occup Environ Health. 2014 Jul-Sep;20(3):194-206. doi: 10.1179/2049396714Y.0000000067.

PMID:

25000107

[**Free PMC Article**](https://www.ncbi.nlm.nih.gov/pubmed/25000107)

[Similar articles](https://www.ncbi.nlm.nih.gov/pubmed?linkname=pubmed_pubmed&from_uid=25000107)

Select item 235059115.

[G protein beta3 subunit **gene** C825T and **angiotensin converting enzyme** **geneinsertion/deletion** polymorphisms in hypertensive Tunisian population.](https://www.ncbi.nlm.nih.gov/pubmed/23505911)

Kabadou IA, Soualmia H, Jemaa R, Feki M, Kallel A, Souheil O, Taieb SH, Sanhaji H, Kaabachi N.

Clin Lab. 2013;59(1-2):85-92.

PMID:

23505911

[Similar articles](https://www.ncbi.nlm.nih.gov/pubmed?linkname=pubmed_pubmed&from_uid=23505911)

Select item 217798036.

[Renin-angiotensin system polymorphisms in relation to **hypertension** status and obesity in a Tunisian population.](https://www.ncbi.nlm.nih.gov/pubmed/21779803)

Mehri S, Mahjoub S, Hammami S, Zaroui A, Frih A, Betbout F, Mechmeche R, Hammami M.

Mol Biol Rep. 2012 Apr;39(4):4059-65. doi: 10.1007/s11033-011-1187-2. Epub 2011 Jul 21.

PMID:

21779803

[Similar articles](https://www.ncbi.nlm.nih.gov/pubmed?linkname=pubmed_pubmed&from_uid=21779803)

Select item 215590527.

[Worldwide spatial **genetic** structure of **angiotensin-converting enzyme** **gene**: a new evolutionary ecological evidence for the thrifty genotype hypothesis.](https://www.ncbi.nlm.nih.gov/pubmed/21559052)

Li X, Sun X, Jin L, Xue F.

Eur J Hum Genet. 2011 Sep;19(9):1002-8. doi: 10.1038/ejhg.2011.66. Epub 2011 May 11.

PMID:

21559052

[**Free PMC Article**](https://www.ncbi.nlm.nih.gov/pubmed/21559052)

[Similar articles](https://www.ncbi.nlm.nih.gov/pubmed?linkname=pubmed_pubmed&from_uid=21559052)

Select item 205807258.

[Genotypic interactions of renin-angiotensin system **genes** with diabetes type 2 in a Tunisian population.](https://www.ncbi.nlm.nih.gov/pubmed/20580725)

Mehri S, Koubaa N, Hammami S, Mahjoub S, Chaaba R, Nakbi A, Zouari B, Abid M, Ben Arab S, Baudin B, Hammami M.

Life Sci. 2010 Jul 3;87(1-2):49-54. doi: 10.1016/j.lfs.2010.05.010. Epub 2010 May 24.

PMID:

20580725

[Similar articles](https://www.ncbi.nlm.nih.gov/pubmed?linkname=pubmed_pubmed&from_uid=20580725)

Select item 204183539.

[Effect of **angiotensin-converting enzyme** **gene** **insertion/deletion** **polymorphism** on steroid resistance in Egyptian children with idiopathic nephrotic syndrome.](https://www.ncbi.nlm.nih.gov/pubmed/20418353)

Saber-Ayad M, Sabry S, Abdel-Latif I, Nabil H, El-Azm SA, Abdel-Shafy S.

J Renin Angiotensin Aldosterone Syst. 2010 Jun;11(2):111-8. doi: 10.1177/1470320309359021. Epub 2010 Apr 23.

PMID:

20418353

[Similar articles](https://www.ncbi.nlm.nih.gov/pubmed?linkname=pubmed_pubmed&from_uid=20418353)

Select item 2041521210.

[Lack of association between renin-angiotensin system (RAS) polymorphisms and **hypertension** in Tunisian type 2 diabetics.](https://www.ncbi.nlm.nih.gov/pubmed/20415212)

Arfa I, Nouira S, Abid A, Bouafif-Ben Alaya N, Zorgati MM, Malouche D, Manai I, Ben Rayana MC, Blousa-Chabchoub S, Ben Romdhane H, Ben Ammar MS, Ben Salah A, Abdelhak S.

Tunis Med. 2010 Jan;88(1):38-41.

PMID:

20415212

[**Free Article**](https://www.ncbi.nlm.nih.gov/pubmed/20415212)

[Similar articles](https://www.ncbi.nlm.nih.gov/pubmed?linkname=pubmed_pubmed&from_uid=20415212)

Select item 2016039811.

[**Angiotensin converting enzyme** D allele is associated with an increased risk of type 2 diabetes: evidence from a meta-analysis.](https://www.ncbi.nlm.nih.gov/pubmed/20160398)

Niu W, Qi Y, Gao P, Zhu D.

Endocr J. 2010;57(5):431-8. Epub 2010 Feb 17.

PMID:

20160398

[**Free Article**](https://www.ncbi.nlm.nih.gov/pubmed/20160398)

[Similar articles](https://www.ncbi.nlm.nih.gov/pubmed?linkname=pubmed_pubmed&from_uid=20160398)

Select item 2005938212.

[**Angiotensin-converting enzyme** **insertion/deletion** **gene** **polymorphism** in a Tunisian healthy and acute myocardial infarction population.](https://www.ncbi.nlm.nih.gov/pubmed/20059382)

Mehri S, Baudin B, Mahjoub S, Zaroui A, Bénéteau-Burnat B, Mechmeche R, Hammami M, Ben Arab S.

Genet Test Mol Biomarkers. 2010 Feb;14(1):85-91. doi: 10.1089/gtmb.2009.0105.

PMID:

20059382

[Similar articles](https://www.ncbi.nlm.nih.gov/pubmed?linkname=pubmed_pubmed&from_uid=20059382)

Select item 1591983413.

[**Angiotensin-converting enzyme** **gene** polymorphisms and obesity: an examination of three black populations.](https://www.ncbi.nlm.nih.gov/pubmed/15919834)

Kramer H, Wu X, Kan D, Luke A, Zhu X, Adeyemo A, McKenzie C, Cooper R.

Obes Res. 2005 May;13(5):823-8.

PMID:

15919834

[**Free Article**](https://www.ncbi.nlm.nih.gov/pubmed/15919834)

[Similar articles](https://www.ncbi.nlm.nih.gov/pubmed?linkname=pubmed_pubmed&from_uid=15919834)

Select item 1509723414.

[**Hypertension**-related **gene** polymorphisms in pre-eclampsia, eclampsia and gestational **hypertension** in Black South African women.](https://www.ncbi.nlm.nih.gov/pubmed/15097234)

Roberts CB, Rom L, Moodley J, Pegoraro RJ.

J Hypertens. 2004 May;22(5):945-8.

PMID:

15097234

[Similar articles](https://www.ncbi.nlm.nih.gov/pubmed?linkname=pubmed_pubmed&from_uid=15097234)

Select item 1464357315.

[Impact of renin-angiotensin-aldosterone system **gene** variants on the severity of **hypertension** in patients with newly diagnosed **hypertension**.](https://www.ncbi.nlm.nih.gov/pubmed/14643573)

Tiago AD, Badenhorst D, Nkeh B, Candy GP, Brooksbank R, Sareli P, Libhaber E, Samani NJ, Woodiwiss AJ, Norton GR.

Am J Hypertens. 2003 Dec;16(12):1006-10.

PMID:

14643573

[Similar articles](https://www.ncbi.nlm.nih.gov/pubmed?linkname=pubmed_pubmed&from_uid=14643573)

Select item 983021016.

[Emergence of Western diseases in the tropical world: the experience with chronic cardiovascular diseases.](https://www.ncbi.nlm.nih.gov/pubmed/9830210)

Forrester T, Cooper RS, Weatherall D.

Br Med Bull. 1998;54(2):463-73. Review.

PMID:

9830210

[Similar articles](https://www.ncbi.nlm.nih.gov/pubmed?linkname=pubmed_pubmed&from_uid=9830210)

Select item 861320317.

[Polymorphisms of renin-angiotensin **genes** among Nigerians, Jamaicans, and African Americans.](https://www.ncbi.nlm.nih.gov/pubmed/8613203)

Rotimi C, Puras A, Cooper R, McFarlane-Anderson N, Forrester T, Ogunbiyi O, Morrison L, Ward R.

**Hypertension**. 1996 Mar;27(3 Pt 2):558-63.

PMID:

8613203

[Similar articles](https://www.ncbi.nlm.nih.gov/pubmed?linkname=pubmed_pubmed&from_uid=8613203)

Select item 864218818.

[**Angiotensin converting enzyme** **gene** **I/D** **polymorphism**, **blood pressure** and the renin-angiotensin system in Caucasian and Afro-Caribbean peoples.](https://www.ncbi.nlm.nih.gov/pubmed/8642188)

Barley J, Blackwood A, Miller M, Markandu ND, Carter ND, Jeffery S, Cappuccio FP, MacGregor GA, Sagnella GA.

J Hum Hypertens. 1996 Jan;10(1):31-5.

PMID:

8642188

[Similar articles](https://www.ncbi.nlm.nih.gov/pubmed?linkname=pubmed_pubmed&from_uid=8642188)

[Back to top](https://www.ncbi.nlm.nih.gov/pubmed/?term=(Hypertension+OR+%E2%80%9Cessential+hypertension%E2%80%9D+OR+%E2%80%9Chigh+blood+pressure+OR+raised+blood+pressure%E2%80%9D)+AND+(%E2%80%9Cangiotensin-converting+enzyme%E2%80%9D+OR+ACEI)+AND+(insertion%2Fdeletion+OR+ACE+I%2FD)+AND+%E2%80%9Cgene+OR+gene+polymorphism%E2%80%9D+AND+Africa*.#result_action_bar)
