# Supplementary material for: Effects of angiotensin converting enzyme gene polymorphism on hypertension in Africa: A meta-analysis and systematic review
Source: PLoS One. 2019 Feb 14;14(2):e0211054. doi: 10.1371/journal.pone.0211054 (PMC6375551; doi:10.1371/journal.pone.0211054)
Supplement: S1 Articles — (DOCX) [file pone.0211054.s003.docx]

Articles used to draw the conclusion

1.Daméhan T, Kologo JK, Karou SD, Yaméogo VN, Bisseye C, Djigma FW et al. Renin-Angiotensin system genes polymorphisms and essential hypertension in Burkina Faso, West Africa. International Hypertens. 2015:979631.

2. [Mary EK](https://www.ncbi.nlm.nih.gov/pubmed/?term=Kooffreh%20ME%5BAuthor%5D&cauthor=true&cauthor_uid=24949289),  [Anumudu](https://www.ncbi.nlm.nih.gov/pubmed/?term=Anumudu%20CI%5BAuthor%5D&cauthor=true&cauthor_uid=24949289) CI, Kumar PL. Insertion/deletion polymorphism of the angiotensin-converting enzyme gene and the risk of hypertension among residents of two cities, South-South Nigeria. [Adv Biomed Res](https://www.ncbi.nlm.nih.gov/pmc/articles/PMC4063107/). 2014; 3: 118.

3.Zarouk WA, Hussein IR, Esmaeil NN, Raslan HM, Reheim HAA, Moguib O et al. Association of angiotensin converting enzyme gene (I/D) polymorphism with hypertension and type 2 diabetes. Bratisl Lek Listy 2012; 113(1):14–18.

4. [H](https://www.ncbi.nlm.nih.gov/pubmed/?term=Soualmia%20H%5BAuthor%5D&cauthor=true&cauthor_uid=23505911)ayet S, [Kabadou IA](https://www.ncbi.nlm.nih.gov/pubmed/?term=Kabadou%20IA%5BAuthor%5D&cauthor=true&cauthor_uid=23505911), [Jemaa R](https://www.ncbi.nlm.nih.gov/pubmed/?term=Jemaa%20R%5BAuthor%5D&cauthor=true&cauthor_uid=23505911), [Feki M](https://www.ncbi.nlm.nih.gov/pubmed/?term=Feki%20M%5BAuthor%5D&cauthor=true&cauthor_uid=23505911), [Kallel A](https://www.ncbi.nlm.nih.gov/pubmed/?term=Kallel%20A%5BAuthor%5D&cauthor=true&cauthor_uid=23505911), [Souheil O](https://www.ncbi.nlm.nih.gov/pubmed/?term=Souheil%20O%5BAuthor%5D&cauthor=true&cauthor_uid=23505911). G protein beta3 subunit gene C825T and angiotensin converting enzyme gene insertion/deletion polymorphisms in hypertensive Tunisian population. [Clin Lab.](https://www.ncbi.nlm.nih.gov/pubmed/23505911) 2013; 59(1-2):85-92.

5. Imen A, Nouira S,  Abid A, Bouafif-Ben NA, Zorgati MM, Malouche D. [Lack of association between renin-angiotensin system (RAS) polymorphisms and hypertension in Tunisian type 2 diabetics](http://www.latunisiemedicale.com/article-medicale-tunisie_1241_en). La tunisie Medicale. 2010; 88(1): 38–41.

6. Naglaa R, Abd-Raboh Neveen, Salah EDH, Manal LL, Samah EB. Association of angiotensin T235 polymorphism with risk of essential hypertension in Egyptian patients. International Journal of Cancer Research. 2012; 8(3):69-82.

7. Ndong AGR, Obame-Engongra LC, Ovono AF. Correlation between the insertion-deletion polymorphism of the angiotensin conversion enzyme gene and hypertension among the Gabonse population. 2018; 12(2): 44-52.

8. Williams SM, Ritchie MD, Phillips JA, Dawson E, Prince M, Dzhura E, Willis A, Semenya A, Summar M, White BC, Addy JH, Kpodonu J, Wong LJ, Felder RA, Jose PA, Moore JH. Multilocus analysis of hypertension: a hierarchical approach. Hum Hered. 2004; 57:28–38.
